# Bioinformatics identification of key genes and therapeutic targets for exercise intervention in polycystic ovary syndrome

**DOI:** 10.3389/fgene.2025.1634588

**Published:** 2025-11-20

**Authors:** Xiaodie Yang, Hong Yang, Dongyi Shen, Qiang Zhang, Chan Zhu

**Affiliations:** 1 Department of Traditional Chinese Medicine, West China Second University Hospital, Sichuan University, Chengdu, China; 2 Key Laboratory of Birth Defects and Related Diseases of Women and Children, Sichuan University, Chengdu, China; 3 Department of Gynaecology, Shanghai municipal Hospital of Traditional Chinese Medicine, Shanghai University of Traditional Chinese Medicine, Shanghai, China; 4 Shanghai University of Traditional Chinese Medicine, Shanghai, China; 5 Department of Sports Medicine, Sichuan Orthopaedic Hospital, Chengdu, Sichuan, China

**Keywords:** polycystic ovary syndrome, exercise, bioinformatics, mechanism, target

## Abstract

**Background and Purpose:**

We aimed to explore the mechanisms and pathways of exercise-based interventions in the treatment of polycystic ovarian syndrome (PCOS).

**Methods:**

In this literature review, studies related to exercise therapy for PCOS that were published in the past 20 years were searched, potentially effective active ingredients were screened, and gene prediction of active ingredients and diseases was conducted using the compound and GeneCards databases, respectively, to identify potential targets of exercised-related bioactive molecules in PCOS. Finally, hub genes and signaling pathways were predicted using bioinformatic methods.

**Results:**

The review identified eight potential effective components were screened out, including irisin, 5α-reductase, kisspeptin, cocaine-and amphetamine-regulated transcript, nerve growth factor, nerve peptide Y, insulin-like growth factor-1, and interleukin-6. A total of 192 target genes for exercise-related components and PCOS were identified, including the hub genes *TNF, IL6, IL1B, JUN, CCND1,* and *PSMA7*.

**Conclusion:**

The hub genes identified in this review indicate that exercise therapy in PCOS may affect the protease system, renin–angiotensin system, inflammatory signal transduction, neuroactive ligand–receptor interaction, and other pathways through the G protein-coupled receptor signaling pathway, neuropeptide signaling pathway, endocrine process, and other biological processes and regulate apoptosis, cell cycle, and intercellular communication.

## 1 Introduction

Polycystic ovarian syndrome (PCOS) is one of the most common reproductive disorders among women of childbearing age. PCOS is a systemic metabolic disease that is characterized by anovulation, infertility, obesity, and hirsutism, and is one of the primary causes of anovulatory infertility in adult women (Dapas and Dunaif, 2022). Exercise is the first-line treatment for PCOS, and meta-analyses have shown that exercise-based interventions can improve menstrual cycles and hirsutism as well as metabolic, body composition, and hormone-related indicators in patients with PCOS (Shan et al., 2021). Moreover, exercise can synergistically improve the efficacy of ovulation-promotion and assisted-reproduction treatments (Hakimi and Cameron, 2017). Furthermore, exercise can improve the body mass index, reproductive function, and hormonal status of patients with PCOS, with fewer adverse reactions and high patient acceptance (Chan et al., 2016). However, the specific mechanism through which exercise improves PCOS remains unclear. The application of bioinformatics is crucial for elucidating underlying mechanisms, as it allows for the systematic integration of fragmented information into a coherent molecular network.

This paper presents the results of a review of clinical and animal experimental studies on exercise therapy for PCOS that have been published over the past 20 years, summarizes the active compounds generated during those exercise interventions for PCOS, and, based on bioinformatics and network topology strategies, explores the main mechanism of action of exercise therapy for PCOS.

## 2 Data and methods

### 2.1 Data sources

Retrieval person and time: The first author conducted the literature search in September 2024.

Time limit for literature search: 2004-09-01 to 2024-09-01.

The PubMed, Embase, Cochrane Library, Web of Science database, CNKI, VIP database, Wanfang Database, and Chinese Biomedical Literature Database were searched.

Search terms: The English search terms included “Polycystic ovarian syndrome,” “Polycystic ovary,” “Stein*Leventhal Syndrome,” “Exercise,” “Training,” and “kinesitherapy.” The search was conducted by combining subject and entry words, and the references of the included articles were traced back to supplement the records that were obtained. The Chinese search terms included “polycystic ovary syndrome,” “polycystic ovary,” “polycystic ovary syndrome,” “exercise,” “training,” and “physical therapy.” Boolean logic words were used for the professional searches.

Manual search: Relevant magazines, conference papers, and materials were manually retrieved.

**FIGURE 1:**
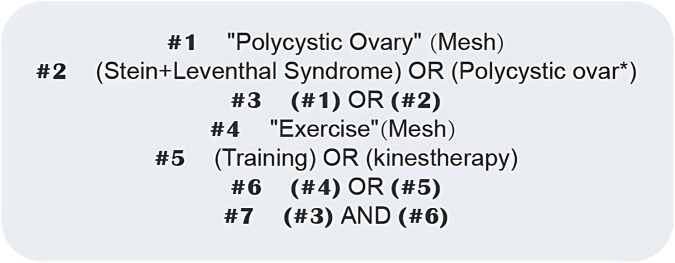
PubMed literature search strategy. Diagram showing the search terms and Boolean operators used for the systematic literature retrieval in PubMed.

Search strategy: The PubMed search mode was undertaken as an example, as shown in Figure 1.

### 2.2 Document inclusion criteria

All relevant studies, including clinical and animal experimental studies as well as observational and interventional research, that involved exercise intervention in PCOS were included without any restriction on the language.

### 2.3 Document exclusion criteria

The exclusion criteria included the following: (1) clinical studies that did not meet the 2003 Rotterdam Standard (Revised, 2003 consensus on diagnostic, 2004) or 2011 Chinese PCOS diagnostic criteria (Linlin and Zijiang, 2011) (e.g., only the ratio of anti-Muller hormone, menstrual cycle, luteinizing hormone (LH)/follicle-stimulating hormone (FSH) was used to diagnose PCOS); (2) animal experiments that did not include a description that confirmed the success of ovarian pathological section; (3) articles that pertained to complex interventions and reported inconclusive primary effects of exercise; (4) articles that did not include effective information on specific compounds/proteins at the mechanistic level; (5) articles that were literature review; (6) master and doctoral dissertations or conference papers; and (7) duplicate detection or duplicate publication.

### 2.4 Literature screening

Using the prespecified inclusion and exclusion criteria, two researchers independently searched the database and selected the appropriate literature; disagreements between researchers were resolved through negotiation, or if no consensus was reached, were resolved by a third researcher.

### 2.5 Data analysis

#### 2.5.1 Screening of protein targets of active ingredients produced by the body after exercise

STITCH is an integrated database of small-molecule chemical interactions with proteins that comprises 390,000 chemicals and 3.6 million proteins from 1,133 organisms (Kuhn et al., 2014). Swiss Target Prediction was used to predict the most likely protein targets of small molecules with a high level of prediction performance (Daina et al., 2019). The SEA database integrates Compound Data Collection (ChEMBL), MDL Drug Data Report (MDDR), and other databases of compound and target information, and calculates compound similarity by using daylight molecular fingerprinting to cluster the targets of similar compounds (Keiser et al., 2007). Targets with a high affinity for the target compound were obtained from the abovementioned database. After combining the search results from the three databases, duplicate values were removed to identify the targets of the active compounds.

#### 2.5.2 Screening of therapeutic targets for PCOS

GeneCards is a comprehensive database of human genes (Rebhan et al., 1997) that contains all annotated and predicted genetic information and integrates approximately 150 gene database resources, including genomic, transcriptomic, proteomic, genetic, clinical, and functional information. In this study, the GeneCards database was searched using the keyword “Polycystic Ovary Syndrome” and the related targets of PCOS were obtained.

#### 2.5.3 Screening of targets related to exercise therapy for PCOS

We proceeded to develop two groups of targets using Venn tools (https://bioinfogp.cnb.csic.es/tools/venny/index.html), which showed the sports-related active ingredient targets with PCOS overlap between the target volume visualizations; the aspects in the overlap constitutes the core target of exercise therapy for PCOS. To clarify the relationship between the active ingredients produced by exercise and key targets, Cytoscape3.10.3 software was used to create a network diagram of the relationship between the active ingredients and targets (Shannon et al., 2003).

#### 2.5.4 Construction of protein interaction network

Protein–protein networks (PPI) were constructed using STRING 11.5 (Szklarczyk et al., 2019). Possible targets of exercise therapy for PCOS were uploaded to the STRING tool to select humans as the species for retrieving the interacting genes/proteins. To clarify the relationship between active compounds and key targets, the search results were imported into Cytoscape3.10.3 software, and the CytoHubba plug-in was used for network topology attribute analysis; the results of multi-dimensions and algorithms were comprehensively compared to screen key genes (hub genes) (Chin et al., 2014).

#### 2.5.5 Gene ontology and enrichment analysis of the kyoto genome encyclopedia

Gene ontology (GO) enrichment analysis (Thomas et al., 2022) and Kyoto Encyclopedia of Genes and Genomes (KEGG) (Kanehisa and Goto, 2000) were used, and the target effects were visualized using WebGestalt (https://www.webgestalt.org/) (Jm et al., 2024) and Hiplot tools (https://hiplot.com.cn/), which provided the basis for further functional research.

## 3 Results

### 3.1 Literature screening results

**FIGURE 2:**
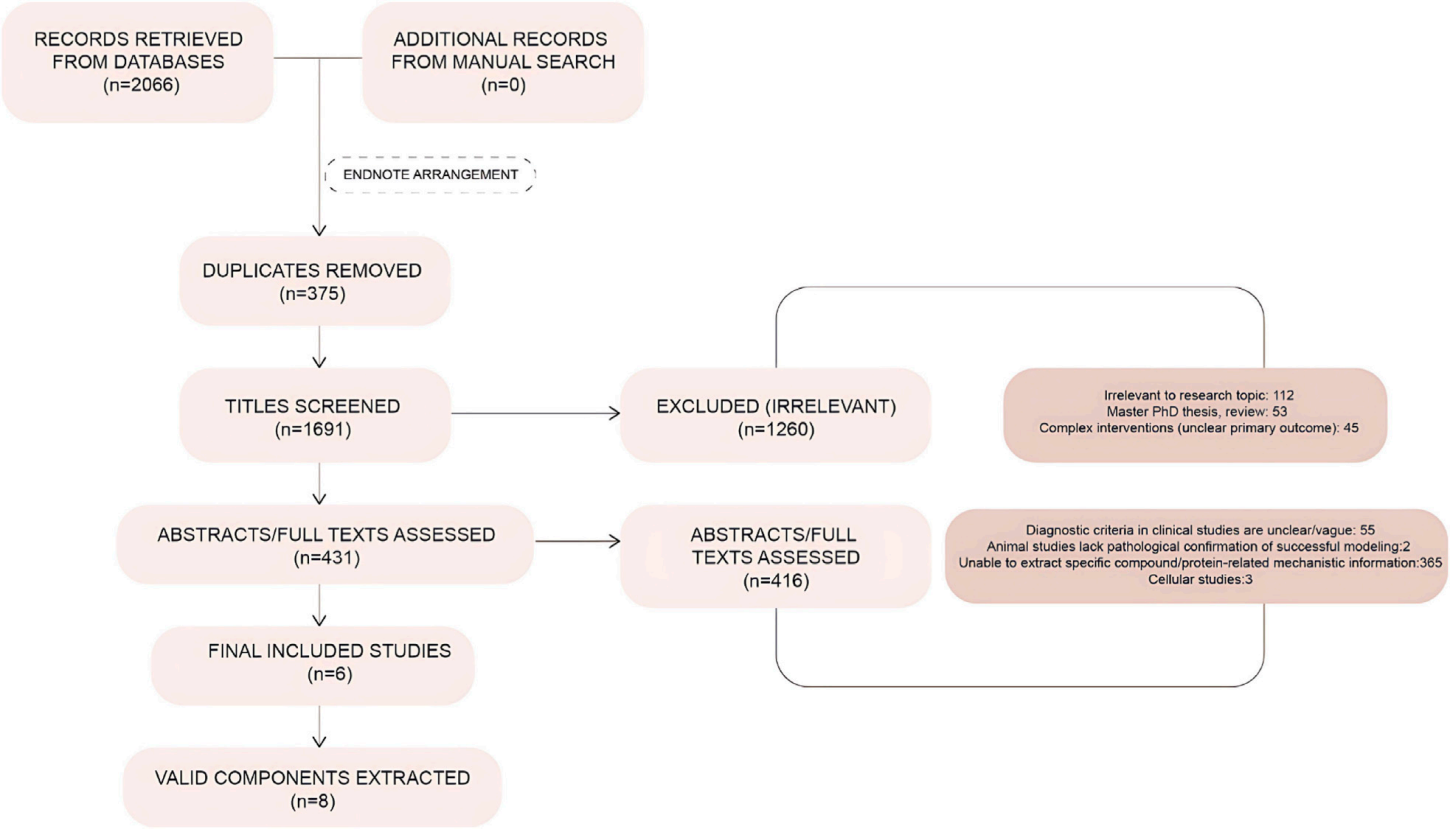
Literature screening flowchart. Flowchart illustrating the process of study identification, screening, and inclusion/exclusion based on predefined criteria.

According to the search terms, 2,066 relevant studies were retrieved, from which irrelevant and duplicate studies were excluded; thus, 1,691 articles were screened by reading the literature. By applying the inclusion and exclusion criteria, 15 articles were selected, all of which were in English. All studies were animal experiments, 6 studies (Table 1) were found to have extractable compounds for subsequent analysis, from which 8 important active ingredients were selected, which were: irisin, 5α reductase, kisspeptin, amphetamine-regulated transcript, cocaine-and amphetamine-regulated transcript (CART), nerve growth factor (NGF), neuropeptide Y (NPY), insulin-like growth factor-1 (IGF-1), and interleukin-6 (IL-6) (Figure 2).

**TABLE 1:** Characteristics of included studies and extracted compounds. Summary of the six included animal studies, detailing the model establishment, exercise intervention, extracted active compounds, and other key characteristics.

Author	Country	Molding method	Movement technique	Research component	Language
Weng et al., (2023)	China	The 3-week-old SD female rats were injected subcutaneously with DHEA for 7 weeks	Treadmill for 1 h a day, 6 days a week for 8 weeks	Irisin	English
Wu et al. (2018)	Brazil	The 3-week-old Wistar magnetic rats were given testosterone propionate injection plus a high-fat diet for 4 weeks	Two hours of weightless swimming, six times a week for 3 weeks	5α-reductase	English
Diane et al. (2015)	Canada	3-week-old JCR: LA-CP female rats spontaneously formed models after 12 weeks of feeding	Voluntary running, 4 h/day, a total of 8 weeks	Kisspeptin, CART	English
Louise et al. (2009)	Sweden	Wistar magnetic rats aged 3 weeks were injected subcutaneously with DHT for 3 months	Exercise wheel free exercise, 3 times/week, a total of 4–5 weeks	NGF,NPY	English
Louise et al. (2008)	Sweden	Wistar magnetic rats aged 3 weeks were injected subcutaneously with DHT for 3 months	Exercise wheel free exercise, 3 times/week, a total of 4–5 weeks	IGF-1,IL-6	English
Luigi et al. (2005)	Sweden	Wistar-kyoto female rats (190–210 g) were molded by one-time intramural injection of estradiol glutarate	Running wheels, chronic voluntary exercise, a total of 5 weeks	NGF	English

CART, cocaine-and amphetamine-regulated transcript, nerve growth factor (NGF), NPY, nerve peptide Y, IGF-1, insulin-like growth factor-1, IL-6, interleukin-6.

### 3.2 Target screening of exercise therapy for PCOS

**FIGURE 3:**
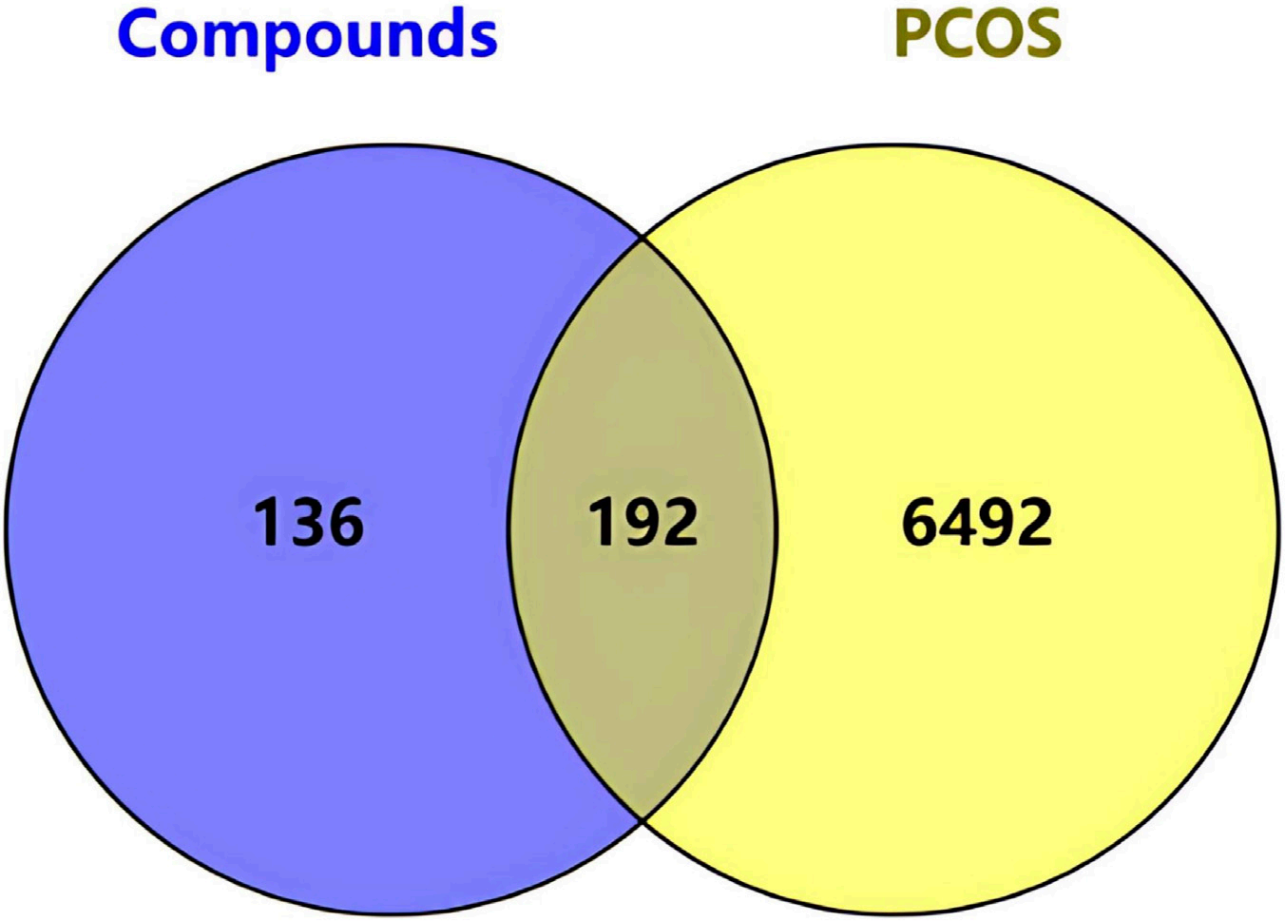
Venn diagram of overlapping targets. Venn diagram showing the intersection between exercise-related compound targets and PCOS-related genes, identifying 192 potential shared targets.

**FIGURE 4:**
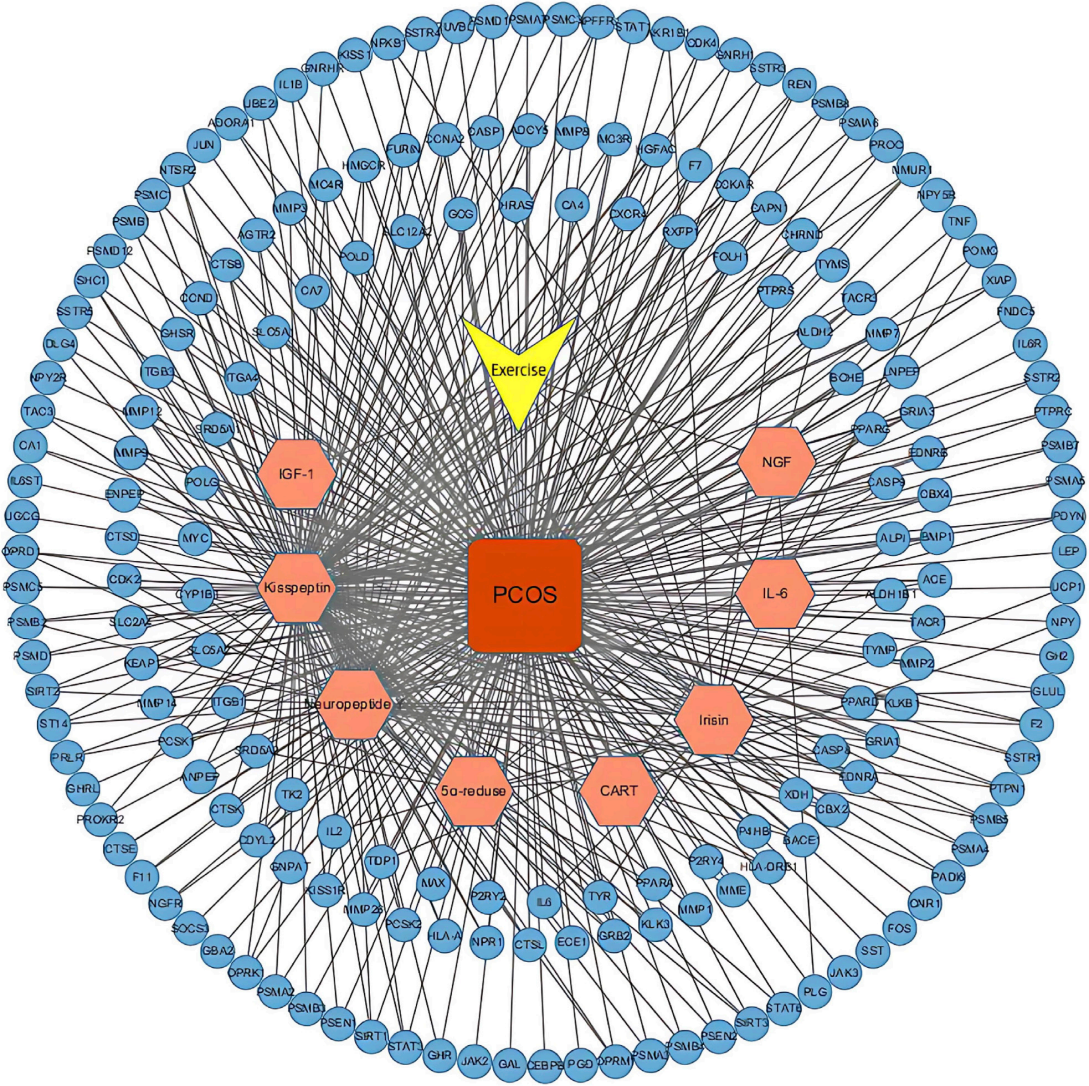
Active ingredient-potential target network. Network visualization depicting the interactions between exercise, the eight active ingredients (orange hexagons), PCOS (red square), and the 192 potential targets (blue circles).

Using the STITCH, SEA, and Swiss Target Prediction databases, 328 genes that interacted with the active ingredients after acupuncture were obtained. 6684 PCOS-related genes were identified from the GeneCards database. After the human gene names in the two original documents were standardized and matched, 192 intersection targets with exercise-active ingredients and PCOS were mapped (Figure 3) to identify the potential therapeutic targets in PCOS. The “motion-active ingredient - target-disease” interaction network is shown in Figure 4, where the yellow triangular arrow represents exercise therapy, the red box represents PCOS, the orange hexagon represents the active ingredient, and the blue circle represents 192 potential therapeutic targets.

### 3.3 Establishment and analysis of the PPI network

**FIGURE 5:**
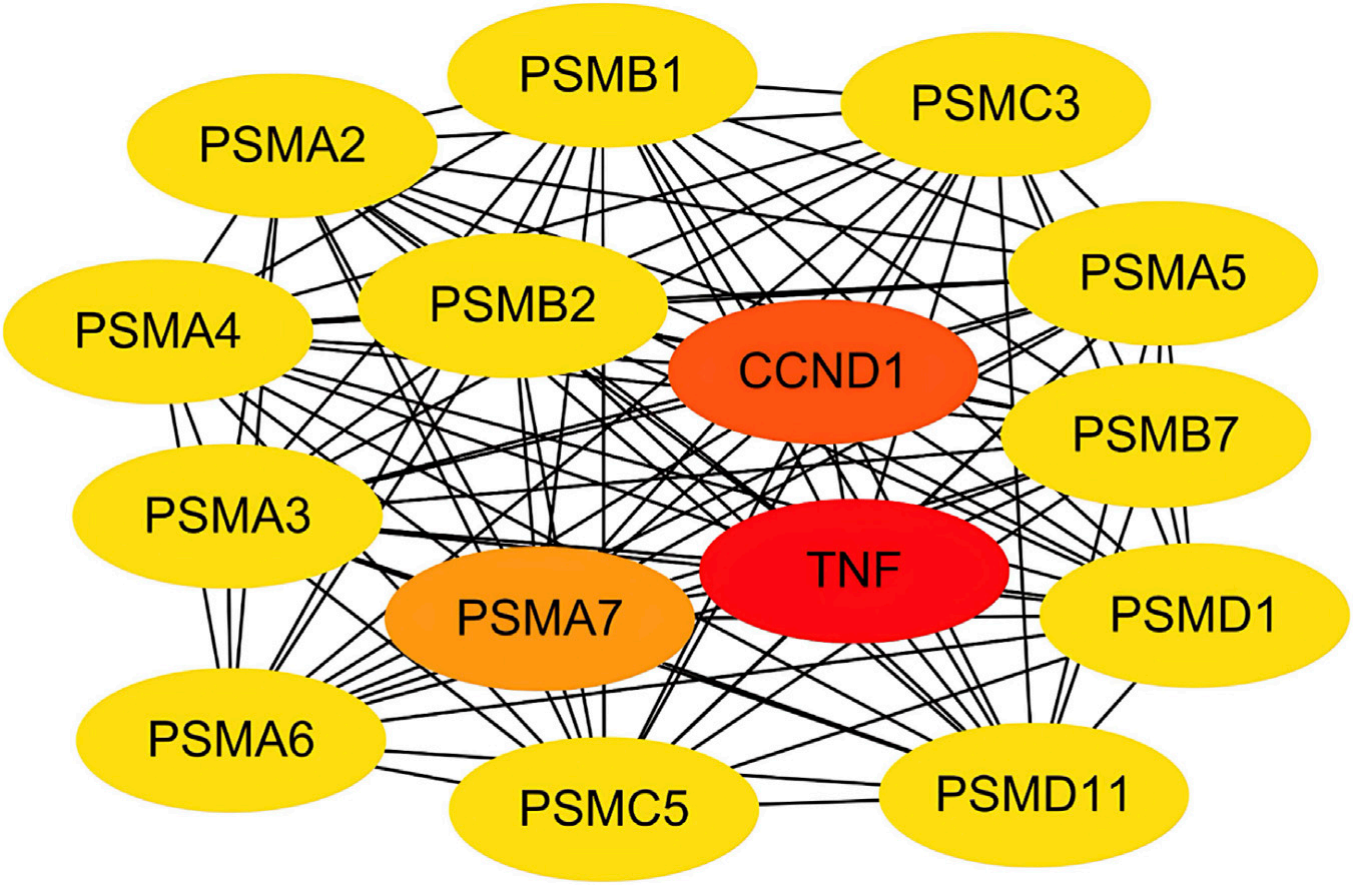
PPI network and hub genes. Protein-protein interaction network of the potential targets. Node color intensity corresponds to topological importance scores calculated by the MCC method, highlighting key hub genes like TNF and IL6.

We constructed a PPI network based on the STRING database and visualized 187 nodes using Cytoscape. The Matthews correlation coefficient (MCC) method is an important iterative formula in the CytoHubba algorithm, providing a more accurate measure of node importance by iteratively calculating degree centrality while accounting for the node’s maximal clique. The topological network algorithm uses the CytoHubba plugin to assign values to each gene and to sort and screen hub genes and subnetworks. It was used to calculate the maximum clique centers of the nodes in the protein interaction network, as shown in Figure 5. *TNF*, *CCND1* and *PSMA7* showed a high correlation degree. In addition, we used various other algorithms to obtain hub genes (Table 2). Among different algorithms, TNF (appeared 7 times) ranked first, followed by *IL6* (6 times), *IL1B* and *JUN* (all appeared 5 times).

**TABLE 2:** Top 10 gene sets from different CytoHubba algorithms. Comparison of the highest-ranked genes identified by seven topological analysis algorithms in CytoHubba, demonstrating consistent hub genes like TNF and IL6 across methods.

Category	Rank methods in CytoHubba						
	EPC	MCC	MNC	Degree	Closeness	BottleNeck	EcCentricity
1	*TNF*	*TNF*	*TNF*	*TNF*	*TNF*	*TNF*	*TNF*
2	*IL1B*	*CCND1*	*IL6*	*IL6*	*IL6*	*IL6*	*IL6*
3	*IL6*	*PSMA7*	*IL1B*	*IL1B*	*IL1B*	*MYC*	*OPRD1*
4	*NFKB1*	*PSMA5*	*NFKB1*	*NFKB1*	*NFKB1*	*GCG*	*NGFR*
5	*STAT3*	*PSMD1*	*STAT3*	*STAT3*	*STAT3*	*JUN*	*PSMA5*
6	*FOS*	*PSMC5*	*MMP9*	*MMP9*	*MMP9*	*CCNA2*	*GHSR*
7	*JUN*	*PSMB2*	*CCND1*	*LEP*	*LEP*	*HLA-A*	*PSMB4*
8	*PPARG*	*PSMC3*	*FOS*	*CCND1*	*FOS*	*UBE2I*	*FOLH1*
9	*CCND1*	*PSMA4*	*LEP*	*FOS*	*JUN*	*OPRM1*	*TYR*
10	*MMP9*	*PSMA3*	*JUN*	*JUN*	*PPARG*	*PDYN*	*IL1B*

### 3.4 GO and KEGG enrichment analysis

**FIGURE 6:**
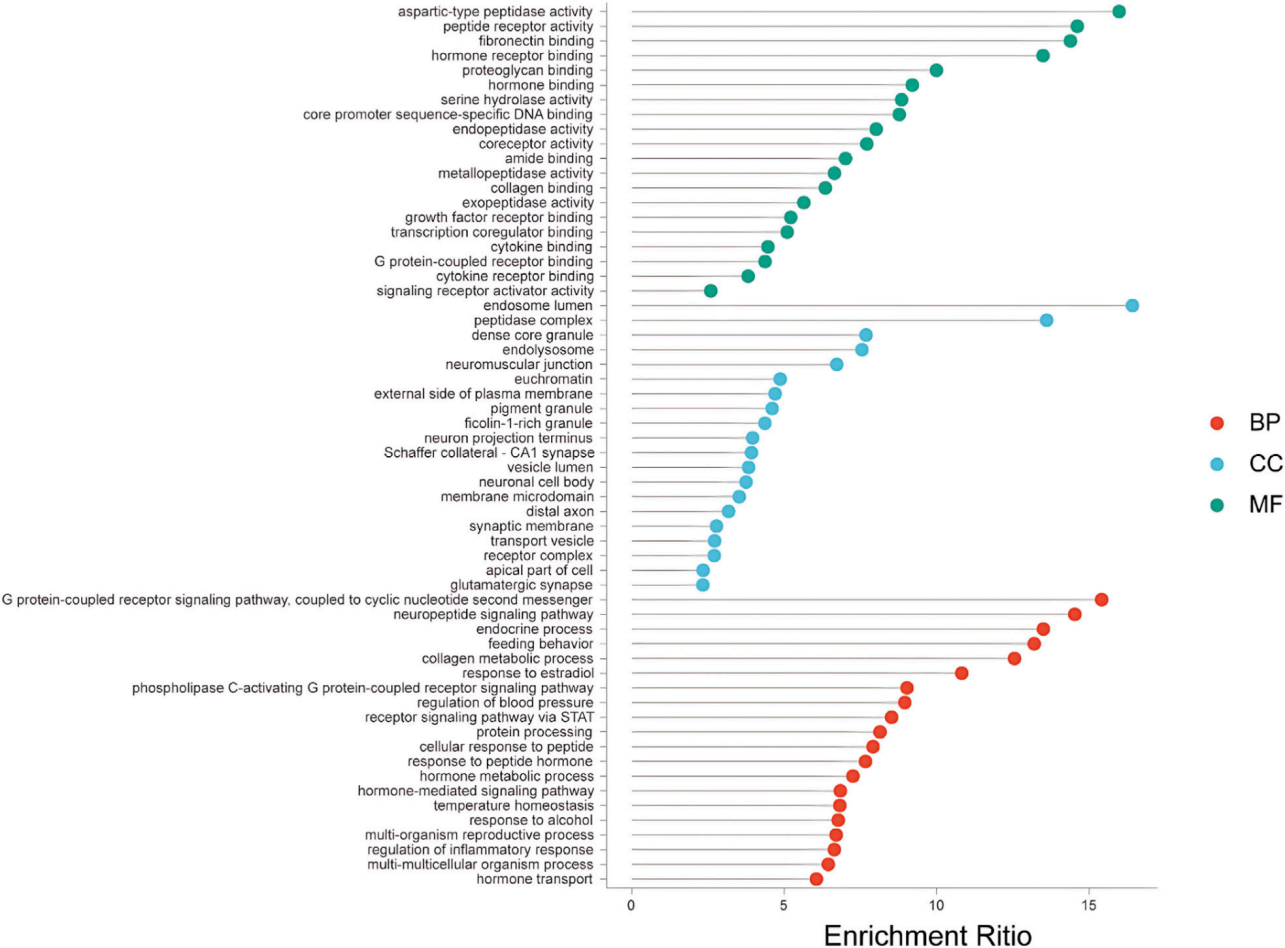
GO enrichment analysis. Bubble chart showing the significant enrichment terms in Biological Process (BP), Cellular Component (CC), and Molecular Function (MF) categories for the potential targets. Bubble size indicates the number of genes, and color indicates the enrichment significance.

GO analysis can reflect the target function from three aspects: cellular components (CC), molecular functions (MF), and biological processes (BP). GO analysis identified 92 CC, 105 MF, and 177 BP. The first 20 CC, MF, BP, and KEGG lists were removed and visualized (Figure 6).

At the BP level, the predicted target was the G protein-coupled receptor signaling pathway, which is mainly involved in the cyclic nucleotide second messenger. Coupled to cyclic nucleotide second messenger, neuropeptide signaling pathway, endocrine processes) and other biological processes have significant significance. At the CC level, the endosome lumen, peptidase complex, and dense core granules occupy a large proportion. The MF is closely associated with aspartic-type peptidase activity, peptide receptor activity, and fibronectin binding.

**FIGURE 7:**
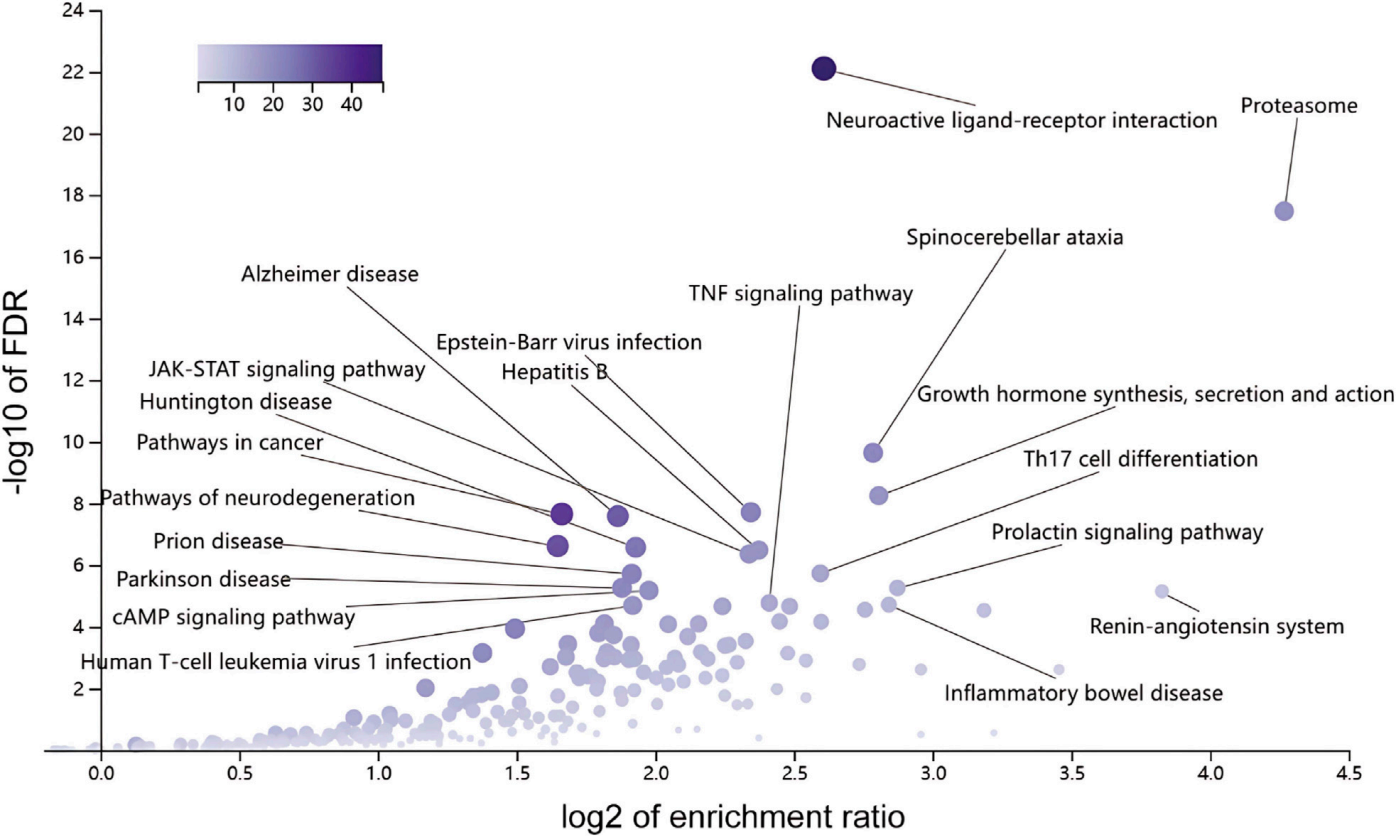
KEGG pathway enrichment analysis. Bar chart displaying the significantly enriched KEGG pathways for the potential targets, suggesting key mechanistic pathways like the Proteasome and Renin-angiotensin system.

The potential KEGG enrichment results are shown in Figure 7. The KEGG signaling pathway involved the Proteasome, Renin-angiotensin system (RAS), prolactin signaling pathway, inflammatory bowel disease, growth hormone synthesis, secretion, and action (growth hormone synthesis, secretion and action, spinocerebellar ataxia, Th17 cell differentiation, neuroactive ligand-receptor interaction, and TNF signaling pathway.

**FIGURE 8:**
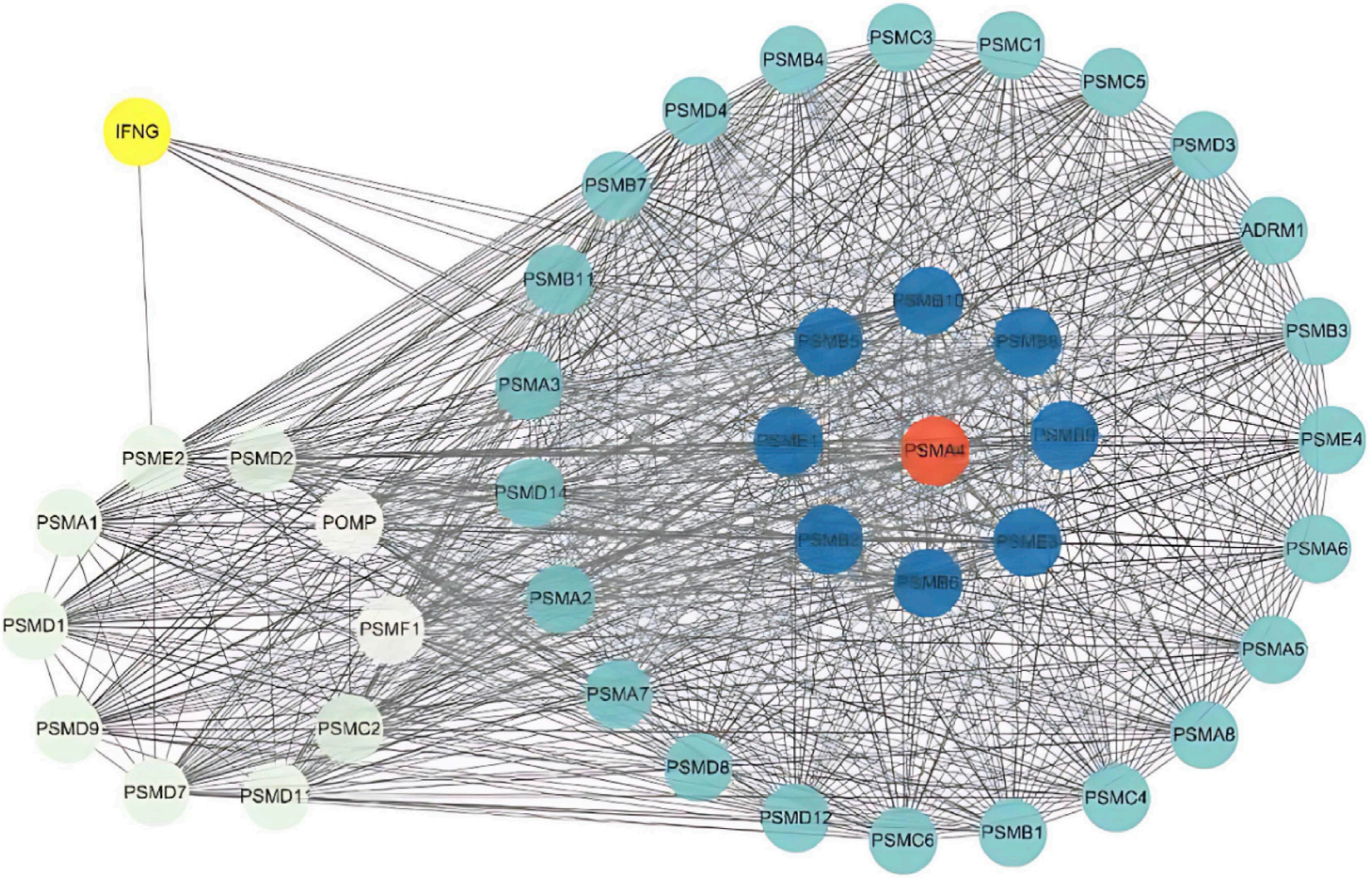
PPI subnetwork for the Proteasome pathway. Interaction subnetwork of proteins involved in the Proteasome pathway, highlighting key proteins like PSMA4 and IFNG.

Figure 8 lists the PPI interaction network shown on KEGG of the proteasome protease system, with 43 related proteins in total, among which PSMA4 and its family members may be the key proteins in this pathway, and the secreted protein IFNG is closely related to the protease system.

## 4 Discussion

Numerous studies have shown that physical exercise has systemic effects, regulates allorgans, and promotes physical health, whereas inactivity can have serious negative effects on health throughout the life cycle (Harridge and Lazarus, 2017). In addition, exercise has potential mental health benefits (Venkatesh et al., 2020). This literature review identified eight potential active ingredients: irisin, 5α-reductase, kisspeptin, CART, NGF, IGF-1 and IL-6. Then, 192 related targets of exercise active ingredients and PCOS were obtained through screening of three compound databases and a comprehensive human gene database, and the key action targets and signaling pathways of exercise therapy for PCOS were predicted using bioinformatics. This study predicted that exercise might regulate the G protein-coupled receptor signaling pathway, neuropeptide signaling pathway, and endocrine processes through key targets such as *TNF*, *IL6*, *IL1B*, *JUN*, *CCND1*, *PSMA7*, etc., thereby affecting the protease, renin-angiotensin, inflammatory signaling, and neuroactive ligand-receptor interaction pathways.

The results of the “exercise-PCOS” co-target PPI network showed that exercise interfered with PCOS and 192 targets, among which TNF, IL-6, IL1B and JUN proteins had relatively strong interactions. *TNF*, *IL-6,* and *IL1B* are associated with various inflammatory disease states and are involved in the regulation of various biological processes, including cell proliferation, differentiation, apoptosis, and lipid metabolism (M Ller and Villiger, 2006). It is well known that chronic inflammation is a typical feature of PCOS, and studies have found that PCOS patients (whether obese or not) have significantly higher levels of circulating inflammatory factors in their blood than healthy women (Nehir et al., 2016), treatment with etanercept, a TNF-α inhibitor, can significantly inhibit androgen increase and follicular dysplasia in letrozole-induced PCOS rats (Lang et al., 2019). Furthermore, moderate exercise has anti-inflammatory effects in various diseases, and meta-analyses have shown that regular exercise can reduce levels of inflammation-related markers in autoimmune diseases, such as C-reactive protein, TNF-α, and IL-6 (Pedersen and Saltin, 2015). Therefore, exercise therapy for PCOS may regulate inflammation and immune status. *JUN* is the protein-coding gene of c-Jun, and c-Jun is the most widely studied protein in the activator protein-1 complex, which is involved in a variety of cellular activities such as proliferation, apoptosis, survival, tumorigenesis, and tissue morphogenesis. c-Jun has always been at the center of molecular networks, with mysterious functional properties that regulate multiple levels of the body in a complex manner. It can crosstalk, amplify, and integrate different signals for tissue development and disease (Qinghang and Xia, 2011). c-Jun N-terminal kinase (JNK) is involved in the regulation of oxidative stress-induced apoptosis in mouse follicular granuloid cells (Qianna et al., 2016). In addition, many studies have shown that under oxidative stress, phosphorylation of JNK in mammalian ovarian granulosa cells increases and the JNK/FoxO1 signaling pathway is activated, leading to apoptosis of ovarian granulosa cells (Xuan et al., 2023) (Kong et al., 2022). These results indicate that phosphorylation of JNK and activation of the JNK/FoxO1 signaling pathway in mammalian ovarian granulosa cells under oxidative stress led to the apoptosis of ovarian granulosa cells. Recent studies have shown that exercise can regulate the mitochondrial unfolded protein response through the JNK signaling pathway to ensure mitochondrial protein stability and function (Gaspar et al., 2023) and that exercise enhances the expression of c-Fos and c-Jun in the hypothalamus, mid-dorsum, and hippocampus (Hong et al., 2014), suggesting that the effect of exercise on JNK may be systemic. Studies have shown that among the various topological analysis methods of the CytoHubba plug-in, the MCC method has better performance in predicting the accuracy of essential proteins and can provide a more comprehensive and accurate evaluation of node centrality (Chicco et al., 2021). Therefore, we also pay special attention to the other two Hub genes predicted by MCC method. *PSMA7* is a protein-coding gene that encodes the alpha subunit of the 20S proteasome core complex, which is involved in protein degradation through the ubiquitin-proteasome pathway and plays an important role in cell proliferation, cell cycle control, transcriptional regulation, immune and stress responses, cell differentiation, and apoptosis. *PSMA7* can play a role in cellular stress by regulating hypoxia-inducing factor-1 α (Du et al., 2009). No studies have found that this gene is related to motility and PCOS; however, a Japanese study suggested that PSMA7 might be involved in the regulation of germ cell survival during spermatogenesis (Shimizu et al., 2014). *CCND1* encodes a highly conserved family of cyclins whose members are characterized by significant periodicity in protein abundance throughout the cell cycle and whose activity is required for cell cycle G1/S transition. *CCND* is misregulated in many cancer types, and the carcinogenic properties of cyclins and cyclin-dependent kinases they activate are well established (Qie and Diehl, 2016). Previous studies have shown that the expression of CCND1 and CCND3 is downregulated in PCOS mouse models (Tong et al., 2024), and may be an important cause of follicular dysplasia in patients with PCOS. At the same time, exercise was found to rejuvenate dormant skeletal muscle stem cells in aged mice by restoring CCND1 activity and inhibiting TNF-β signaling (Brett et al., 2020). In summary, the abovementioned genes are closely related and affect each other in the process of exercise therapy for PCOS, and are concentrated in the four major phenotypes of inflammation, apoptosis, oxidative stress, and the cell cycle, which are worthy of further exploration.

The results of GO analysis showed that the mechanism of action of exercise therapy for PCOS is a multi-target, multi-layer approach and involves multiple biological processes. First, BP levels affect the G protein-coupled receptor and neuropeptide signaling pathways. NPY is a neurotransmitter widely distributed in the central nervous system and plays an important role in the regulation of animal physiology and behavior. They constitute the largest and most diverse neuronal messengers that mediate neuroendocrine signaling and extrasynaptic communication in the nervous system. The secreted neuropeptides mainly bind to G protein-coupled receptors on neighboring neurons or distant target cells. To regulate various physiological processes and brain functions, such as eating, sleep, arousal, reproduction, and learning (Jiang et al., 2024). Studies on *C. elegans* have found that hermaphrodite-specific neurons release neuropeptides that cause them to dramatically increase their movement before laying eggs, affecting some reproductive behaviors in response to environmental influences (Hardaker et al., 2001). Hypothalamic neurons expressing NPY and agoutine chromoprotein-related peptides (NPY/AgRP neurons) are closely associated with fertility regulation. Selective activation of NPY/AgRP neurons significantly improves the frequency of hypothalamic gonadotropin-releasing hormone (GnRH)/LH pulses in patients (Coutinho et al., 2019). Kisspeptin is a neuropeptide produced by the KISS1 gene. Altered kisspeptin signal transduction can lead to abnormal GnRH pulse secretion that induces an elevated LH/FSH ratio, which leads to PCOS progression. Studies have shown that both swimming (Arisha and Moustafa, 2019) and running (Kacar et al., 2023) can inhibit kisspeptin secretion, and thereby inhibit GnRH secretion to improve reproductive function. In addition, BP suggested that exercise intervention in PCOS is related to the improvement of the biological process of endocrine. PCOS, in most patients, is complicated by insulin resistance and related hyperinsulinemia, and this phenomenon is prevalent even in non-obese patients with PCOS. Hyperandrogenism and insulin resistance have a bidirectional relationship, which forms a vicious cycle in the endocrine status of patients with PCOS (Moghetti and Tosi, 2021). Exercise training is considered an effective stimulus for improving insulin resistance (Sun and Ding, 2020). In insulin resistance, the adaptive response to exercise training induces an improvement in glucose tolerance and enhanced sensitivity of skeletal muscle insulin to glucose transport. Although the specific mechanism has not been fully elucidated, recent studies have suggested that it may be closely related to the upregulation of specific components of the glucose transport system in muscles and the alleviation of mitochondrial dysfunction (Mthembu et al., 2022; Siemers et al., 2023).

The results of the KEGG analysis showed that exercise interfered with PCOS mainly in the protease system. The proteasome has a wide range of functions that can cleavecell proteins into peptides and play a regulatory role in various biological processes. Previous studies showed that exercise improves proteasome function and promotes synaptic plasticity in mice with brain injury, and this mechanism may be related to improved oxidative stress levels (Szabo et al., 2010). Recent studies have suggested that exercise can enhance hippocampal proteasome activity, and thereby contribute to neurogenesis. This benefit is reversed after the use of the proteasome inhibitor MG132, which suggests that exercise can significantly enhance the activity of the proteasome system in the central nervous system (Niu et al., 2020). Currently, there is a lack of direct evidence regarding the relationship between the protease system and PCOS. However, PCOS is associated with insulin resistance and obesity. In obese mouse models, immunoglobulin superfamilies containing leucine-rich repeats (Islr) control ubiquitin-independent proteasome degradation of INα by interacting with *Pmsa4*, a key gene in the protease system, and regulating insulin sensitivity (Zhang et al., 2023). In addition, although no direct evidence has been found in human studies, the effects of exercise and physical exercise on the RAS of the second enrichment pathway have been confirmed in various animal models of diseases (Gomes-Santos et al., 2014; Ren et al., 2016; Liu et al., 2022; Chang et al., 2022). Physical exercise can benefit from the regulation of the ACE-AngII-AT1 axis of the RAS (Nunes-Silva et al., 2017). In addition to genes related to proliferation, some genes enriched in the prolactin signaling pathway, such as *JAK2*, *NFKB1*, *PRLR*, *SHC1*, *SOCS3*, *STAT1*, and *STAT3,* are related to cell–cell interaction communication, and this suggests that exercise may affect reproductive system function through the exercise-nervous system via cell–cell communication via neurohumoral signaling.

## 5 Limitation

Only eight representative active substances produced after exercise were included in this study, and this research was limited by the current research level, which is characterized by a lack of human data research. The selection of model animals in the included literature as well as the exercise mode and duration were not completely uniform, which may have caused certain interference in the results of the analysis.

Nevertheless, this review employed bioinformatics and network topology analyses to explore the biological mechanisms of exercise therapy for PCOS, and the insights from this research provide a basis for future experimental research.
